# Predictive and diagnostic value of MCP-1, MIF, and ICAM-1 in Type-2 diabetes mellitus patients with diabetic kidney disease

**DOI:** 10.12669/pjms.41.7.12283

**Published:** 2025-07

**Authors:** Yingying Wang, Gang Cheng, Gouqin Wang, Xiaochun Zhou, Min Ma, Jianqin Wang

**Affiliations:** 1 Yingying Wang Department of Nephrology, The Second Hospital & Clinical Medical School, Lanzhou University; The Second Hospital & Clinical Medical School, Key laboratory of Nephropathy, The Second Hospital & Clinical Medical School, Lanzhou University, Nephropathy Clinical Medical Research Center, The Second Hospital & Clinical Medical School, Lanzhou University, Lanzhou 730000, Gansu, People’s Republic of China; 2 Gang Cheng Department of Nephrology, The Second Hospital & Clinical Medical School, Lanzhou University, The Second Hospital & Clinical Medical School, Lanzhou University, Lanzhou 730030, Gansu, People’s Republic of China; 3 Gouqin Wang Department of Nephrology, The Second Hospital & Clinical Medical School, Lanzhou University; The Second Hospital & Clinical Medical School, Key laboratory of Nephropathy, The Second Hospital & Clinical Medical School, Lanzhou University, Nephropathy Clinical Medical Research Center, The Second Hospital & Clinical Medical School, Lanzhou University, Lanzhou 730000, Gansu, People’s Republic of China; 4 Xiaochun Zhou Department of Nephrology, The Second Hospital & Clinical Medical School, Lanzhou University; The Second Hospital & Clinical Medical School, Key laboratory of Nephropathy, The Second Hospital & Clinical Medical School, Lanzhou University, Nephropathy Clinical Medical Research Center, The Second Hospital & Clinical Medical School, Lanzhou University, Lanzhou 730000, Gansu, People’s Republic of China; 5 Min Ma Department of Nephrology, The Second Hospital & Clinical Medical School, Lanzhou University, Lanzhou 730000, Gansu, People’s Republic of China; 6 Jianqin Wang Department of Nephrology, The Second Hospital & Clinical Medical School, Lanzhou University; The Second Hospital & Clinical Medical School, Key laboratory of Nephropathy, The Second Hospital & Clinical Medical School, Lanzhou University, Nephropathy Clinical Medical Research Center, The Second Hospital & Clinical Medical School, Lanzhou University, Lanzhou 730000, Gansu, People’s Republic of China

**Keywords:** Diabetes kidney disease, Intercellular adhesion molecule-1, Macrophage migration inhibitory factor, Monocyte chemoattractant protein-1, Type-2 diabetes mellitus

## Abstract

**Objective:**

To explore the predictive value of monocyte chemoattractant protein-1 (MCP-1), macrophage migration inhibitory factor (MIF), and intercellular adhesion molecule-1 (ICAM-1) in patients with Type-2 diabetes mellitus (T2DM) complicated by diabetic kidney disease (DKD).

**Methods:**

This cross-sectional retrospective study included T2DN patients admitted to the Nephrology Department of Lanzhou University Second Hospital from September, 2022 to March, 2024. DKD was assessed by measuring the ratio of urinary albumin to creatinine. A Receiver Operating Characteristic (ROC) analysis was performed to evaluate the predictive value of MCP-1, MIF, and ICAM-1 for DKD.

**Results:**

A total of 241 patients were included, predominantly 158 males (65.6%), with a median age of 60 (53-69) years. Sixty-seven patients had no DKD (normal proteinuria), while 174 patients presented with DKD; of them, 95 cases had microalbuminuria, and 79 cases had high proteinuria. The MCP-1, MIF, and ICAM-1 levels in the high-proteinuria group were significantly higher than in other groups (all P<0.05). The multivariate logistic regression analysis showed that high levels of MCP-1, MIF, and ICAM-1 are risk factors for the development of DKD. ROC analysis demonstrated that the area under the curve (AUC) of MCP-1, MIF, and ICAM-1 for diagnosing DKD were 0.895 (95% CI: 0.857-0.933, P<0.001), 0.719 (95% CI: 0.653-0.785, P<0.001), and 0.827 (95% CI: 0.773-0.880, P<0.001), respectively. The combined prediction of DKD by the three factors was 0.941 (95% CI: 0.914-0.968, P<0.001).

**Conclusions:**

MCP-1, MIF, and ICAM-1 are risk factors for developing DKD. A combination of these indexes may have a good predictive value for DKD.

## INTRODUCTION

Diabetic kidney disease (DKD) is a common and serious microvascular complication of Type-2 diabetes mellitus (T2DM)^1^ and is the leading cause of end-stage renal disease, which seriously affects the health and quality of life of patients with diabetes.^1^,^2^ With the global incidence of diabetes on the rise, the increasing prevalence of DKD is associated with a heavy socio-economic burden.^3^,^4^ At present, while the clinical treatment of diabetes has markedly improved, the pathogenesis of DKD has not been fully clarified.^3^-^5^ Therefore, in-depth research on the mechanisms of DKD has important clinical significance.^4^-^7^ In recent years, the research has focused on the role of inflammatory response in the pathogenesis of DKD and has identified several crucial factors.^8^-^12^ Macrophage chemoattractant protein-1 (MCP-1) plays an essential role in the recruitment and aggregation of inflammatory cells. It can attract inflammatory cells such as macrophages to migrate towards kidney tissue, thereby triggering local inflammatory reactions and disrupting the normal structure and function of the kidney.^8^ Macrophage migration inhibitory factor (MIF) regulates macrophage function, participates in the network regulation of various cytokines, plays a central role in the inflammatory cascade, and is closely related to inflammation and fibrosis progression in DKD.^9^,^10^ Intercellular adhesion molecule-1 (ICAM-1) mainly mediates intercellular adhesion, promotes the adhesion of inflammatory cells to vascular endothelial cells, infiltrates tissues, and plays an essential role in the formation of the local kidney inflammatory microenvironment.^11^,^12^

Multiple studies have shown that MCP-1, MIF, and ICAM-1 are associated with the occurrence and development of various kidney diseases.^8^-^10^ However, their specific role in the pathogenesis of DKD is not yet fully understood. This study aimed to explore the predictive value of MCP-1, MIF, and ICAM-1 in patients with T2DM complicated by DKD.

MCP-1 contributes to renal inflammation by recruiting macrophages, MIF accelerates fibrosis through activating MAPK pathways, and ICAM-1 promotes leukocyte adhesion and infiltration, exacerbating kidney damage. Understanding these detailed mechanisms underscores their potential as biomarkers for DKD.^11^,^12^

## METHODS

This retrospective study used a cross-sectional survey method to select T2DN patients admitted to the Nephrology Department of Lanzhou University Second Hospital from September 2022 to March 2024. The urinary albumin-to-creatinine ratio (UACR) was calculated, and the diagnosis of DKD was made based on the Guidelines for the Prevention and Treatment of diabetes Nephropathy in China (2021).^13^ Based on the diagnosis, the patients were divided into the non-DKD [normal proteinuria (UACR<30 mg/g)] and the DKD group. Patients in the DKD group were further divided into microalbuminuria (UACR 30-300 mg/g) and macroproteinuria (UACR>300 mg/g) subgroups.

Sample size calculation was based on previous studies with expected AUC values around 0.85, a significance level (α) of 0.05, and statistical power of 90%. A minimum sample size of approximately 210 participants was determined adequate.

### Ethical approval:

The study was approved by the ethics committee of the Second Hospital & Clinical Medical School of Lanzhou University approved this study with the number 2024A-368; dated: March 21, 2024.

### Inclusion criteria:

- Clear history of T2DM.
- Age 18 and above.
- Complete clinical data.

### Exclusion criteria:

- Type-1 diabetes mellitus, monogenic diabetes syndrome or other special types of diabetes.
- Kidney disease caused by any other reason.
- Obvious infection.
- Ketoacidosis.
- Use of immunosuppressants or glucocorticoids within the past six months.
- Malignant tumors, hematological and autoimmune diseases.
- Pregnant or lactating women.

The recorded general, clinical and laboratory data included gender, age, height, weight, systolic blood pressure (SBP), diastolic blood pressure (DBP), duration of T2DM, UACR, 24-hours urinary protein content (24hUTP), glycated hemoglobin (HbA1c), blood urea nitrogen (BUN), serum creatinine (Scr), uric acid (UA), triglycerides (TG), total cholesterol (TC), high-density lipoprotein cholesterol (HDL-C), low-density lipoprotein cholesterol (LDL-C).

MCP-1, MIF, and ICAM-1 levels were measured in the serum of fasting venous blood by enzyme-linked immunosorbent assay. ELISA kits were obtained from Sinovac Biotech (Beijing), with sensitivities of 2.5 pg/ml (MCP-1), 10 ng/L (MIF), and 5 ug/L (ICAM-1). The intra-assay and inter-assay coefficients of variation were less than 5% and 10%, respectively. Body mass index (BMI) was calculated using the following formula: BMI = weight/(height)^2^. The estimated glomerular filtration rate (eGFR) was calculated according to the CKD-EPI formula.^14^

### Statistical analysis:

SPSS 27.0 statistical software (IBM Corp., Armonk, NY, USA) was used for data analysis. Continuous data that conformed to a normal distribution (according to the Kolmogorov-Smirnov test) was described as mean ± standard deviation (SD), and the analysis of variance was used to evaluate the differences between the groups. The LSD method was initially used for pairwise comparison. To control for multiple comparisons and reduce Type I errors, a Bonferroni correction was subsequently applied, adjusting the significance threshold accordingly. Continuous data with skewed distribution was described as the median (upper and lower quartiles), and the Kruskal Wallis H test was used to determine the differences between the three groups. The Nemenyi test was used for pairwise comparison.

The categorical data is represented as n (%) and analyzed using the chi-square test. Risk factors of urinary albumin were analyzed by multivariate logistic regression analysis. GraphPad Prism 9 (GraphPad Software Inc., San Diego, CA, USA) was used to draw receiver operating characteristic (ROC) curves. The area under the curve (AUC) was calculated to evaluate the predictive value of MCP-1, MIF, and ICAM-1 for DKD and to calculate sensitivity, specificity, and critical values. P<0.05 was considered statistically significant.

## RESULTS

A total of 241 T2DM patients [158 males (65.6%)] were included in this study, with a median age of 60 (53-69) years. Among them, 67 patients comprised the non-DKD group, and 174 patients comprised the DKD group. Of the DKD patients, there were 95 cases of microalbuminuria and 79 cases of macroproteinuria. There was no statistically significant difference in gender, age, SBP, DBP, duration of T2DM, BUN, TG, HDL-C, and LDL-C among the groups (all *P*>0.05). UACR, 24hUTP, SCR, MCP-1, MIF, and ICAM-1 levels gradually increased in the normal proteinuria, microalbuminuria, and macroalbuminuria groups. In contrast, eGFR levels gradually decreased, with significant differences among the three groups (all *P*<0.05). The BMI of the microalbuminuria group and the macroalbuminuria group were significantly higher than that of the normal proteinuria (*P*<0.05). The HbA1c and UA of the group with high urinary albumin levels were significantly higher than those with low urinary albumin levels and those with normal urinary albumin (*P*<0.05) (Table-I).

**Table-I:** Characteristics of Patients.

Variables	Non-DKD (n = 67)	DKD (n = 174)		P
	Normal proteinuria (n = 67)	Microproteinuria (n = 95)	Macroproteinuria (n = 79)	
Male (yes), n(%)	44 (65.7)	60 (63.2)	54 (68.4)	0.772
Age (years)	61(54-70)	61(53-71)	58(51-66)	0.119
BMI (kg/m²)	23.952.99	25.363.07^a^	25.123.23^a^	0.014
SBP(mm/Hg)	135.61±22.94	141.85±21.51	140.71±20.16	0.172
DBP(mm/Hg)	79(70-89)	82(73-94)	86(77-93)	0.055
Duration of T2DM (years)	10(6-15)	10(3-15)	10(5-15)	0.376
UACR	16.14(9-21.86)	98.52(79.07-130.37)^a^	394.74(347.25-476.19)^ab^	<0.001
24hUTP (g/24h)	0.18(0.09-0.25)	0.27(0.13-0.66)^a^	0.5(0.21-1.46)^ab^	<0.001
HbA1c (%)	8.3(7.2-9.9)	8.4(7-10.7)	9.3(7.8-11.5)^a^	0.048
BUN (mmol/L)	5.6(4.65-7.1)	6.1(5.2-7.9)	6.3(4.9-7.5)	0.111
SCR (μmol/L)	70(57-86)	79(65-97)^a^	107(86-159.9)^ab^	<0.001
UA (mmol/L)	303(266-368)	342(277-405)	351(305-392)^a^	0.012
TG (mmol/L)	1.56(1.08-2.34)	1.64(1.28-2.32)	1.6(1.16-2.38)	0.593
HDL-C (mmol/L)	1.14(0.93-1.3)	1.13(0.92-1.34)	1.12(0.93-1.35)	0.876
LDL-C (mmol/L)	2.91(1.93-3.43)	2.67(2.14-3.32)	2.91(2.22-3.4)	0.574
eGFR	83.02±27.33	74.48±29.48^a^	66.53±20.77^ab^	0.001
MCP-1 (pg/ml)	312.43±42.43	353.43±49.42^a^	387.48±65.27^ab^	<0.001
MIF (ng/L)	116.19±38.72	146.16±47.23^a^	166.96±58.96^ab^	<0.001
ICAM-1 (ug/L)	285.42(259.29-352.76)	362.81(306.52-396.98)^a^	473.36(386.93-564.81)^ab^	<0.001

***Note:*** BMI, body mass index; SBP, systolic blood pressure; DBP, diastolic blood pressure; T2DM, Type-2 diabetes mellitus; UACR, urinary albumin-to-creatinine ratio; 24hUTP, 24-h urinary protein quantity; HbA1C, glycated hemoglobin; BUN, blood urea nitrogen; Scr, serum creatinine; UA, uric acid; TG triglycerides; HDL-C, high-density lipoprotein cholesterol; LDL-C, low-density lipoprotein cholesterol; eGFR, estimated glomerular filtration rate; MCP-1, monocyte chemoattractant protein-1; MIF, macrophage migration inhibitory factor; ICAM-1, intercellular adhesion molecule-1.

a *P* < 0.05 compared with Normal proteinuria;

^b^*P* < 0.05 compared with Microproteinuria.

Logistic regression analysis with urinary albumin as the dependent variable (with=1, none=0) and MCP-1, MIF, and ICAM-1 as independent variables showed that higher levels of MCP-1 (OR=1.040, 95% CI: 1.025-1.055, *P*<0.001), MIF (OR=1.013, 95% CI: 1.004-1.023, *P*=0.006) and ICAM-1 (OR=1.012, 95% CI: 1.006-1.019, *P*<0.001) were risk factors for DKD in T2DM (Table-II).

**Table-II:** Multivariate logistic regression analysis.

Variable	B	S.E	Waldχ^2^	P	OR	95% CI
MCP-1 (pg/ml)	0.039	0.007	28.55	<0.001	1.040	1.025-1.055
MIF (ng/L)	0.013	0.005	7.535	0.006	1.013	1.004-1.023
ICAM-1 (ug/L)	0.012	0.003	15.504	<0.001	1.012	1.006-1.019

***Note:*** B indicates partial regression system; *S.E.* indicates standard error; *Wald χ^2^=*(B/*S.E.*)^2^; *OR* is odds ratio; *95%CI* is the confidence interval of *OR;* MCP-1, monocyte chemoattractant protein-1; MIF, macrophage migration inhibitory factor; ICAM-1, intercellular adhesion molecule-1.

**Fig.1:**
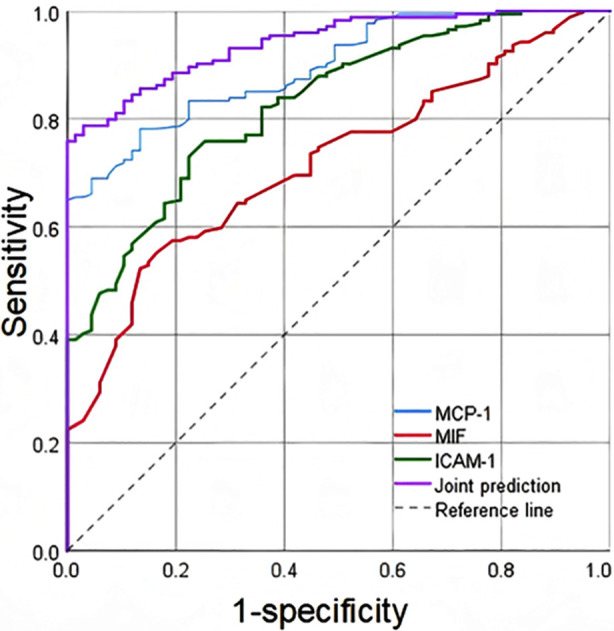
ROC of MCP-1, MIF, and ICAM-1 predicting DKD in T2DM. MCP-1, monocyte chemoattractant protein-1; MIF, macrophage migration inhibitory factor; ICAM-1, intercellular adhesion molecule-1.

The microproteinuria and macroproteinuria groups were then combined, and the patients were divided into the non-DKD (n=67) and DKD (n=174) groups. ROC curves which were done to explore the clinical predictive value of MCP-1, MIF, and ICAM-1 for DKD in T2DM showed that the AUC of MCP-1, MIF, and ICAM-1 for diagnosing DKD were 0.895 (95% CI: 0.857-0.933, *P*<0.001), 0.719 (95% CI: 0.653-0.785, *P*<0.001), and 0.827 (95% CI: 0.773-0.880, *P*<0.001), respectively. The combination of the three factors has the highest value in predicting DKD in T2DM patients, with a AUC of 0.941 (95% CI: 0.914-0.968, *P*<0.001) (Fig.1, Table-III).

**Table-III:** Predictive Value of MCP-1, MIF, and ICAM-1 for DKD in T2DM.

Variable	AUC	95%CI	Cut off	Youden index	Sensitivity	Specificity	P
MCP-1 (pg/ml)	0.895	0.857-0.933	352.575	0.649	0.649	1.000	<0.001
MIF (ng/L)	0.719	0.653-0.785	162.18	0.389	0.523	0.866	<0.001
ICAM-1 (ug/L)	0.827	0.773-0.880	334.67	0.506	0.73	0.776	<0.001
Joint prediction	0.941	0.914-0.968			0.759	1.000	<0.001

***Note:*** MCP-1, monocyte chemoattractant protein-1; MIF, macrophage migration inhibitory factor; ICAM-1, intercellular adhesion molecule-1; T2DM, Type-2 diabetes mellitus; AUC, area under the curve.

## DISCUSSION

This study aimed to investigate the predictive value of MCP-1, MIF, and ICAM-1 for DKD in T2DM patients. High MCP-1, MIF, and ICAM-1 levels were identified as risk factors for DKD in T2DM with a certain predictive value, especially when combined.

Potential confounding factors, such as genetic background, dietary patterns, medication adherence, and socioeconomic factors, were not fully accounted for in our study. These variables may significantly influence MCP-1, MIF, and ICAM-1 expression levels. Future studies should include detailed assessment and control of these confounders through rigorous study design, comprehensive data collection, and advanced statistical methods such as multivariate adjustment and propensity score matching.

Previous studies have shown that MCP-1 plays a key role in the pathogenesis of DKD. As DKD worsens, MCP-1 levels continue to rise and positively correlate with the degree of renal macrophage infiltration.^15^,^16^ Raina et al.^17^ showed that MCP-1 gene polymorphism can affect its expression level, altering the susceptibility to DKD. The elevated levels of MCP-1 in this study reflect its crucial role in the inflammatory cascade response induced by hyperglycemia. However, due to differences in genetic background, lifestyle, and environmental factors among populations in different regions, there may be subtle variations in the magnitude and specific pathways that regulate MCP-1 levels.^18^,^19^ For instance, European and American populations have a higher intake of fat and protein, which may indirectly affect the expression of MCP-1 by affecting metabolic pathways.^18^ Similarly, the genetic susceptibility of Asian populations may make MCP-1 expression in this population more sensitive to certain stimuli.^19^

Xing et al.^20^ found that under high glucose conditions, MIF can activate the MAPK signaling pathway in renal mesangial cells, promote cell proliferation and extracellular matrix synthesis, and accelerate the process of renal fibrosis. An animal model study by Khalilpour et al.^21^ showed that knockout of the MIF gene can significantly reduce the degree of renal lesions in diabetic mice. Liu et al.^22^ demonstrated the increase in the expression of MIF in stimulated or damaged renal tissue, indicating a direct correlation with the severity of renal injury. The results of this study corroborate these observations, further confirming the essential role of MIF in the pathogenesis of DKD. However, it is important to note that differences in MIF detection methods, types of experimental models, and model establishment methods among different studies may lead to certain biases in evaluating MIF effects. Moreover, different detection antibodies with varying affinities for MIF may cause fluctuations in the detection results.

ICAM-1 is an acute phase protein marker of inflammation.^11^ The high expression of ICAM-1 on the surface of renal endothelial cells can lead to increased leukocyte adhesion, trigger local inflammatory reactions, and accelerate kidney damage.^11^ The clinical study by Siddiqui et al.^23^ showed that ICAM-1 levels indicate microvascular complications in T2DM patients. A study by Huang et al.^24^ found that specific mutations in the ICAM-1 gene are closely related to the progression rate of DKD. This study also showed that the levels of ICAM-1 in the microalbuminuria and the macroalbuminuria groups were significantly higher than those in the normal proteinuria group. These results further confirm that ICAM-1 is involved in the inflammation and injury process of DKD.

Compared with previous studies conducted in diverse populations, our findings regarding elevated MCP-1, MIF, and ICAM-1 align well with those reported in European and North American cohorts.^15^,^17^,^23^ However, variations in detection methods, genetic predispositions, lifestyle, and disease management practices could explain minor discrepancies. Further cross-population comparative studies are required to clarify the universality and variability of these biomarkers’ predictive capacities.

Currently, a kidney biopsy is the gold standard for diagnosing DKD. However, due to its invasiveness and high cost, some patients may refuse or have biased test results due to comorbidities.^25^ Therefore, using a simple blood test to screen for DKD is very attractive. The ROC analysis results of this study showed that the AUC of MCP-1 alone was 0.895, while combining MCP-1 with MIF and ICAM-1 increased the AUC to 0.941. Therefore, the combination of the three biomarkers has a certain predictive value for the occurrence of DKD in T2DM patients. In summary, these findings provide valuable references for evaluating biomarkers such as MCP-1, MIF, and ICAM-1 in T2DM patients. Dynamic monitoring of changes in MCP-1, MIF, and ICAM-1 levels can help evaluate the progression of DKD and guide the adjustment of clinical treatment plans. Clinically, these biomarkers offer potential advantages over traditional diagnostic methods such as kidney biopsy, given their non-invasive nature and simplicity. These findings suggest that integrating MCP-1, MIF, and ICAM-1 measurement into clinical practice could enhance early detection and management of DKD, although prospective validation is required.

### Limitations:

This is a single-center, cross-sectional, retrospective study with a small sample size. The single-center nature in Northwest China limits generalizability; future multi-center studies including diverse populations are recommended. Insufficient consideration of all potential confounding factors, such as genetic factors, dietary patterns, psychosocial factors, and healthcare disparities, may affect the robustness and generalizability of the results. The study used single-point MCP-1, MIF, and ICAM-1 measurements, with limited external validity, and a lack of in-depth mechanism research. In the future, more extensive and diverse population studies are needed to validate the results and comprehensively evaluate the role of MCP-1, MIF, and ICAM-1 dysregulation in the progression of DKD.

## CONCLUSION

This study indicates that MCP-1, MIF, and ICAM-1 have a certain predictive value for DKD in T2DM patients and can serve as potential therapeutic targets. However, various factors, such as race, living environment, testing methods, and research design, may have affected the research results. Further multi-center, large-sample studies covering different ethnic groups are needed to explore the mechanisms of MCP-1, MIF, and ICAM-1 effect in patients with DKD. Such studies will provide a reference for the early identification of DKD risk in T2DM patients. Despite promising predictive value, practical implementation in clinical settings requires consideration of cost-effectiveness, standardized assays, and prospective validation to confirm improvement in patient outcomes.

### Authors’ contributions:

**YW and GC:** Literature search, Study design and manuscript writing.

**GW, XZ, MM and JW:** Data collection, data analysis and interpretation. Critical Review.

**YW and GC:** Manuscript revision and validation and are responsible for the integrity of the study.

All authors have read and approved the final manuscript.
